# EFFECTS OF BEING FORGOTTEN ON ADULT CHILDREN OF MOTHERS WITH DEMENTIA

**DOI:** 10.1093/geroni/igad104.2593

**Published:** 2023-12-21

**Authors:** Kristie Wood, Marie-Anne Suizzo

**Affiliations:** Colorado University, Anschutz Medical School, Denver, Colorado, United States; UT Austin, Austin, Texas, United States

## Abstract

Mothers with dementia sometimes do not recognize their adult children. Yet, research on the effects of being forgotten by a mother with dementia is scarce. Object relations theorists posit that healthy development of a child’s sense of self is linked to being remembered by a primary caretaker. Further, being held in mind by a primary caregiver allows the child to form a cohesive identity. It is unclear how adult children’s identities are impacted by being forgotten by their mothers. To investigate this phenomenon, this qualitative study explored the effects of being forgotten on adult children in the context of dementia. The aims were: 1) to develop an in-depth understanding of what it means for adults to be forgotten by their mothers with dementia, and 2) to gain insight on the effect of being forgotten on adults’ identities. Data were gathered through 12 interviews. Data was analyzed following an interpretative phenomenological analysis approach. Preliminary findings suggest adult children of mothers with dementia experience being forgotten as both a threat to their identity and forewarning of continued poor disease outcomes. The following themes illustrate this: 1) The sense that one’s existence was not important enough to be remembered, 2) Feelings of failure due to the inability to spark recognition of themselves in their mother’s mind, and 3) Dread for the disease progression. While research regarding adult children of parents with dementia largely focuses on practical aspects of caregiving, clinical implications of these findings suggest that this population suffers silently from being forgotten.

